# Involvement of the mouth and jaw area in dermatological diseases

**DOI:** 10.3205/dgkh000560

**Published:** 2025-06-17

**Authors:** Vasudevi Ramiah, Shenbaga Lalitha Sankar, Karthik Shunmugavelu, Sajid Tajamul Hussain, Manju Palanisamy Sadasivam, Shyam Sundar Behura

**Affiliations:** 1 Department of Dental Surgery, Governement Medical College and Hospital, Krishnagiri, Tamil Nadu, India; 2 Department of Biochemistry, Tagore Medical College and Hospital, Rathinamangalam, Chennai, Tamil Nadu, India; 3 Department of Dentistry, PSP medical college hospital and research institute Tambaram Kanchipuram main road Oragadam Panruti Kanchipuram district Tamil Nadu, India; 4 Department of Periodontology and Implantology, Sree Balaji, Dental College and Hospital, Chennai, Tamil Nadu, India; 5 Department of Research, Meenakshi Academy of Higher Education and Research (MAHER-Deemed to be University), Chennai, Tamil Nadu, India; 6 Department of Oral & Maxillofacial Pathology, Kalinga Institute of Dental Sciences, Kalinga Institute of Industrial Technology (KIIT), Deemed to be University, Bhubaneswar, Odisha. India

**Keywords:** oral mucosal lesions, dermatological disease, association, prevalence

## Abstract

**Background:**

The oral cavity can be affected by a variety of disorders, and many systemic disorders have wide range of manifestations in the oral cavity. Oral mucosal lesions can be early manifestations of the disease or the only symptom of dermatological diseases; therefore no symptom or sign in the oral cavity should be neglected.

**Materials and methods:**

1,131 patients who came to the Oral and Maxillofacial Pathology department for various dermatological treatments were included. The demographic details were obtained from each patient and a thorough dermatological examination was done. On examining the oral cavity, the size and the site of any lesions were noted. The results were entered in SPSS version 21 and descriptive statistics calculated (p<0.05 was considered statistically significant).

**Results:**

Out of 1,131 patients, 237 patients had both with dermatological and oral mucosal lesions. Psoriasis (44.3%) was most frequently accompanied by lesions of the oral mucosa, followed by pemphigus (31.2%) and bullous pemphigoid (10.1%). The most common site of involvement in the oral cavity was the palate (38.3%). Patients in the age group of 25–50 years (76%) were affected more than the other age groups. Male (49.7%) and female (50.3%) patients were equally affected.

**Conclusion:**

Diagnosing oral lesions in dermatology practice and mucocutaneous lesions in dental practice can play a pivotal role in patient management. Thus, comprehensive knowledge is necessary to diagnose these cases in dental and dermatology practice.

## Zusammenfassung

**Hintergrund:**

Die Mundhöhle kann von einer Vielzahl von Erkrankungen betroffen sein, und viele systemische Erkrankungen haben ein breites Spektrum der Manifestation in der Mundhöhle. Läsionen der Mundschleimhaut können frühe Hinweise der Manifestation oder das einzige Symptom der dermatologischen Erkrankungen sein, daher sollte jedes Symptom in der Mundhöhle nicht vernachlässigt werden.

**Methode:**

1.131 Patienten, die sich wegen verschiedener dermatologischer Behandlungen vorstellten, wurden in die Studie aufgenommen. Die demografischen Daten wurden erhoben, und es wurde eine umfassende dermatologische Untersuchung durchgeführt. Bei der Untersuchung der Mundhöhle wurden die Größe und die Stelle der Läsion notiert. Die Ergebnisse wurden in SPSS Version 21 eingegeben und die deskriptive Statistik durchgeführt (p<0,05 wurde als statistisch signifikant betrachtet).

**Ergebnisse:**

237 der 1.131 Patienten hatten parallel zu dermatologischen Symptomen Läsionen der Mundschleimhaut. Psoriasis (44,3%) war am häufigsten von Läsionen der Mundschleimhaut begleitet, gefolgt von Pemphigus (31,2%) und bullösem Pemphigoid (10,1%). Die häufigste Lokalisation in der Mundhöhle war der Gaumen (38,3%).

Patienten in der Altersgruppe 25–50 Jahre (76%) waren häufiger betroffen als die anderen Altersgruppen. Männliche (49,7%) und weibliche (50,3%) Patienten waren gleichermaßen betroffen.

**Schlussfolgerung:**

Die Bedeutung der Diagnose oraler Läsionen spielt in der dermatologischen Praxis und von mukokutanen Läsionen in der zahnärztlichen Praxis eine zentrale Rolle bei der Behandlung. Das muss bei der Diagnose dieser Fälle in der zahnärztlichen und dermatologischen Praxis entsprechend berücksichtigt werden.

## Introduction

It is well known that oral health reflects overall health. This statement is also true in many cases of dermatological disorders where oral lesions occur along with or precede skin lesions. Dermatological diseases are systemic pathoses which, apart from the skin, manifest in other regions, e.g., the oral cavity, eyes, nails, and hair. This enables the dentist to observe the case well before systemic manifestations have occurred [1]. Although the oral lesions are usually benign, progress of the dermatological component might negatively influence the patient’s quality of life. Early diagnosis and treatment benefit the patient. Most of the oral lesions that occur concurrently with dermatological disorders have been referred to as Oral Mucosal Lesions (OML) that manifests in various forms, such as patches, plaques, bullae, blisters or ulcers, to distinguish them from infections. Some of dermatological lesions are strongly associated with oral lesions and could be overlooked by dentists due to a lack of awareness [2]. This study was done to find the prevalence of OML among patients who attended a tertiary dermatology clinic and determine possible correlations with their cutaneous counterparts.

## Materials and methods

The study included patients treated for dermatological diseases. Demographic details were obtained from each patient and a thorough dermatological examination was done with histopathological confirmation. The study group was divided into age groups of 0-25 years, 26–50 years and 51–60 years. OML were assessed as abnormal changes such as swelling, plaque, fissures or a patch that are visible on the oral mucosa. While examining the oral cavity, the size and site of the lesion were noted. The lesions were histopathologically examined to confirm the diagnosis. The results were entered in SPSS version 21, and descriptive statistics were calculated, with statistical significance set at p<0.05. The study procedure was explained to the patients in detail, and informed consent was subsequently obtained. Confidentiality of the patients was maintained. The participants were informed about their oral conditions, and health education was provided. Approval of the study was obtained from the Institutional Ethical Board, Institutional Review Board (IRB), and patient consent as per the IRB guidelines wase also obtained for each part of this study.

## Results

1,131 patients with dermatological diseases were examined. Of these, 237 patients had both dermatological and OML. Patients were afflicted with pemphigus vulgaris, lichen planus, psoriasis, bullous pemphigoid, xeroderma pigmentosum, discoid lupus erythematosus, rhinophyma, vitiligo, erythema multiforme, lichenoid reaction, candidiasis, acanthosis nigricans and psoriasiform dermatitis associated with the respective cutaneous counterparts (Table 1 (Tab. 1)). The lesions were asymptomatic and were observed upon intraoral examination.

### Gender distribution

Of the patients with dermatological diseases, 562 (49.7%) were male and 569 (50.3%) were female.No gender difference was found among patients with parallel OML (Table 2 (Tab. 2) and Table 3 (Tab. 3)). 

### Age dependency and symptoms

In terms of psoriasis, pemphigus vulgaris and bullous pemphigoid, the majority of the patients belonged to the age group of 30–40 years. The major cutaneous manifestation of psoriasis was silvery keratotic scales. The most common clinical type of pemphigus vulgaris was reticular, and buccal mucosa was mostly involved, with more females affected. The skin lesions showed hypertrophic plaque form, and dystrophic nails were observed. Clinically, oral lesions precede skin lesions in many cases and appear as blisters which rupture rapidly, resulting in painful erosions.

One case of discoid lupus erythematosus was observed at a 72-year-old male patient. An ulcerative oral lesion was observed on the lips. The extra-oral manifestation was a butterfly-shaped malar rash and discoid rash, but no other systemic manifestations were seen. Histopathologically, the lesion was characterized by hyperparakeratosis, focal areas of liquefaction, and degeneration of the basal layer.

The oral manifestation of lichen planus was characterized by white striae and a streak or patch pattern in buccal mucosa.

### Site distribution of oral mucosal lesions

21.5% of the lesions were located on the buccal mucosa, 11.8% were found on the gingiva, 38.4% on the palate, 18.1% onthe lips and 10.1% were found involving the tongue. 

## Discussion

The study highlighted dermatological lesions that were associated with OML. OML may occur either before or after dermatological lesions and might have therapeutic consequences.

### Psoriasis

According to Hernández-Pérez et al. [2], oral lesions were found in 67.5% of patients with psoriasis. This was similar to our finding, which showed 52 out of 105 cases of psoriasis had a strong positive correlation. Tomb et al. [3] conducted a study on 400 psoriasis patients and 1,000 controls, revealing a significant correlation between psoriasis and fissured tongue (33.2% vs. 9.9%, P<0.0001) and geographic tongue (7.7% vs. 1%, P<0.0001). Notably, fissured tongue was highly prevalent in pustular psoriasis (83.3% vs. 30% in other forms). While these oral features are strongly associated with psoriasis, they are not pathognomonic. The prevalence of fissured tongue was higher than previously reported, and patients were often unaware of these signs. Hernández-Pérez et al. [2] and Tomb et al. [3] suggest a strong association between psoriasis and oral lesions, particularly fissured and geographic tongue. However, these features are not pathognomonic, and many patients remain unaware of them. They also found a high incidence of HLA-DR7 in patients with psoriasis and EM, a finding that has not yet been explored in our population, highlighting the need for further research to understand its clinical significance.

### Pemphigus vulgaris (PV)

Similar to our findings, Dagistan et al. [4] stated that oral lesions were the first to manifest. Buccal mucosa, lips, and soft palate are most involved. As the oral cavity is subject to trauma during mastication, the thin roof of the blister ruptures easily and forms an erosion or ulcer in the area. As reported in the literature, our patients also presented with the two most common symptoms related to PV, that is, pain and burning sensation. Many cases of PV have been reported to begin as generalized lesions involving multiple intraoral sites, as in our case, in which the patient developed lesions on the buccal mucosa and tongue. While the buccal mucosa has been reported to be one of the most commonly affected sites, the tongue, which was also affected in our case, is a rare site for PV [5].

### Bullous pemphigoid

All cases showed oral manifestations, which was similar to Budimir et al. [6], who had also reported oral lesions in all the cases of systemic cases of bullous pemphigoid. The oral manifestations initially started as bullae/vesicles which ruptured, leaving a raw, eroded, ulcerative area. It involved the buccal mucosa, tongue and lips. Onset of bullae followed by rupture and leaving out painful ulcerations [2], [6]. 

### Discoid lupus erythematosus

In our study, a single case (0.4%) of discoid lupus erythematosus (DLE) was observed in a 72-year-old male, presenting with an ulcerative oral lesion on the lips and extra-oral manifestations of a butterfly-shaped malar rash and discoid rash, without systemic involvement. Histopathological findings included hyperparakeratosis, focal liquefaction, and basal layer degeneration. Among 23 patients with DLE skin manifestations, only 1 (4.3%) exhibited both skin and oral involvement, indicating the rarity of oral lesions in DLE. Oral lesions may be present in each of two types of lupus erythematosus. Oral manifestations of discoid lupus erythematosus are referred as “oral discoid lesions” and they occur in about 20% of patients [7]. These may occur without involvement of skin lesions or before the skin lesions develop. Oral discoid lesions most commonly occur on the labial mucosa, vermillion border and buccal mucosa.

Typical cases of oral discoid lesions are characterized clinically by the presence of white papules, central erythema, a border zone of irradiating white striae and peripheral telangiectasia [8]. This was similar to the findings of the study by Schmidt et al. [9], who also reported initial oral manifestation of this disorder.

### Lichen planus

From our study population we found that lichen planus was observed in 18 patients (7%), with a higher prevalence in males (66.7%) compared to females (33.3%). Among 78 patients with lichen planus having skin manifestations, only 18 (23%) exhibited both skin and oral manifestations, indicating that oral involvement was less common. Oral manifestations of white striae/streak or patch pattern in buccal mucosa were similar to the observations by Thete et al. [10].Those authors observed 19 cases of lichen planus, of which 7 lesions were plaque-like, 9 lesions exhibited papules, and 3 lesions had an ulcerated appearance; the sites involved included 17 buccal mucosa cases and 2 labial mucosa cases. This finding was in line with Eisen et al. [11], who stated that the disease manifests in the oral cavity several weeks before the skin lesions. About 15% of oral lichen planus patients have concurrent skin lesions.

### Erythema multiforme

In our study, one case of erythema multiforme was observed in a male patient, whereas Goncalves et al. [12] found erythema multiforme (n=21) mainly among women (62%) between the ages of 20 and 40 years (48%). Only 7 patients presented oral lesions, and of this number, 57% reported a relation between the disease and viral infections, especially herpes simplex, 29% reported a relation with drugs like Azathioprine, Thalidomide etc. which has the negative impacts such as blood in the urine or stool, unusual bruising, fatigue, development of mouth sores and ulcers, and 14% did not mention any kind of relation. As for lesion type, 43% presented ulcerated lesions on the buccal mucosa, followed by lips (43%) and ulcerations in the tongue (24%).

### Xeroderma pigmentosum

In our study, xeroderma pigmentosum was observed in one female patient and not in any male patients. Whereas Wayli et al. [13] reported an equal incidence in males and females with a history of consanguinity. Clinical symptoms usually manifest on regions exposed to sunlight. Individuals with XP develop cancer of the skin caused by exposure to mutational effects of radiant energy. Therefore, the disease is considered both hereditary and environmental; oral signs and symptoms of this disease are rare. Malignancies such as squamous cell carcinoma can develop on various parts of the oral mucosa but in our case, the patient did not have any signs of malignancy [14].

### Psoriasiform dermatitis

Suliman et al. [15] stated that the psoriasiform reaction pattern accounted for 56.7%, whereas in our study 9 cases were observed, 3 in males and 6 in females. All cases showed oral manifestations. 

## Conclusion

The oral cavity can be affected by various disorders, and many systemic diseases manifest through oral lesions or mucosal changes. Any symptom in the oral cavity should not be overlooked, as it may serve as an early indicator of an underlying disease. In this study, the number of patients with oral mucosal lesions in dermatological conditions was relatively low. However, the most notable finding was the frequent association of psoriasis with oral manifestations, followed by pemphigus and lichen planus. Histopathological findings support the diagnosis, with psoriasis-associated oral lesions showing parakeratosis and acanthosis, pemphigus lesions exhibiting intraepithelial clefting and acantholysis, and lichen planus characterized by lichenoid inflammation and basal layer degeneration. Recognizing such oral manifestations in dental and dermatological practice is crucial for early detection and management of these conditions.

The significance of diagnosing these oral lesions in dermatology practice and mucocutaneous lesions in dental practice plays a pivotal role in patient management. Thus, comprehensive knowledge is required to diagnose these cases in dental and dermatology practice, and such a multidisciplinary therapeutic approach will improve the patient’s prognosis. This study also shows that diagnosis and management of these oral lesions should also be carried out by oral clinicians in order to improve oral health functioning during the course of the disease. Intraoral examinations should be incorporated into routine dermatological examinations, as the oral manifestations can represent preliminary signs or can coexist with the diseases. The oral mucous membrane alone may be involved, but is very often overlooked by dermatologists, dentists, ENT specialists, and physicians. Primary health care providers should be able to manage such cases to quickly start appropriate interventions for the benefit of the patients, without delay or specialist consultations. Future studies need to focus on the size of the population suffering from a given disease, which may be useful to identify the statistical correlation between oral manifestations and dermatological diseases.

## Notes

### Ethical approval

Institutional Ethical Board, Institutional Review Board (IRB) approval, patient consent as per the IRB guidelines were obtained for each part of this study.

### Funding

None. 

### Authors’ ORCIDs

- Ramiah V: https://orcid.org/0009-0009-1493-772X
- Sankar SL: https://orcid.org/0000-0002-5855-7974
- Shunmugavelu K: https://orcid.org/0000-0001-7562-8802
- Hussain ST: https://orcid.org/0000-0003-0028-0711
- Sadaisavam MP: https://orcid.org/0009-0005-8791-3009
- Behura SS: https://orcid.org/0000-0001-7281-0318

### Competing interests

The authors declare that they have no competing interests.

## Figures and Tables

**Table 1:**
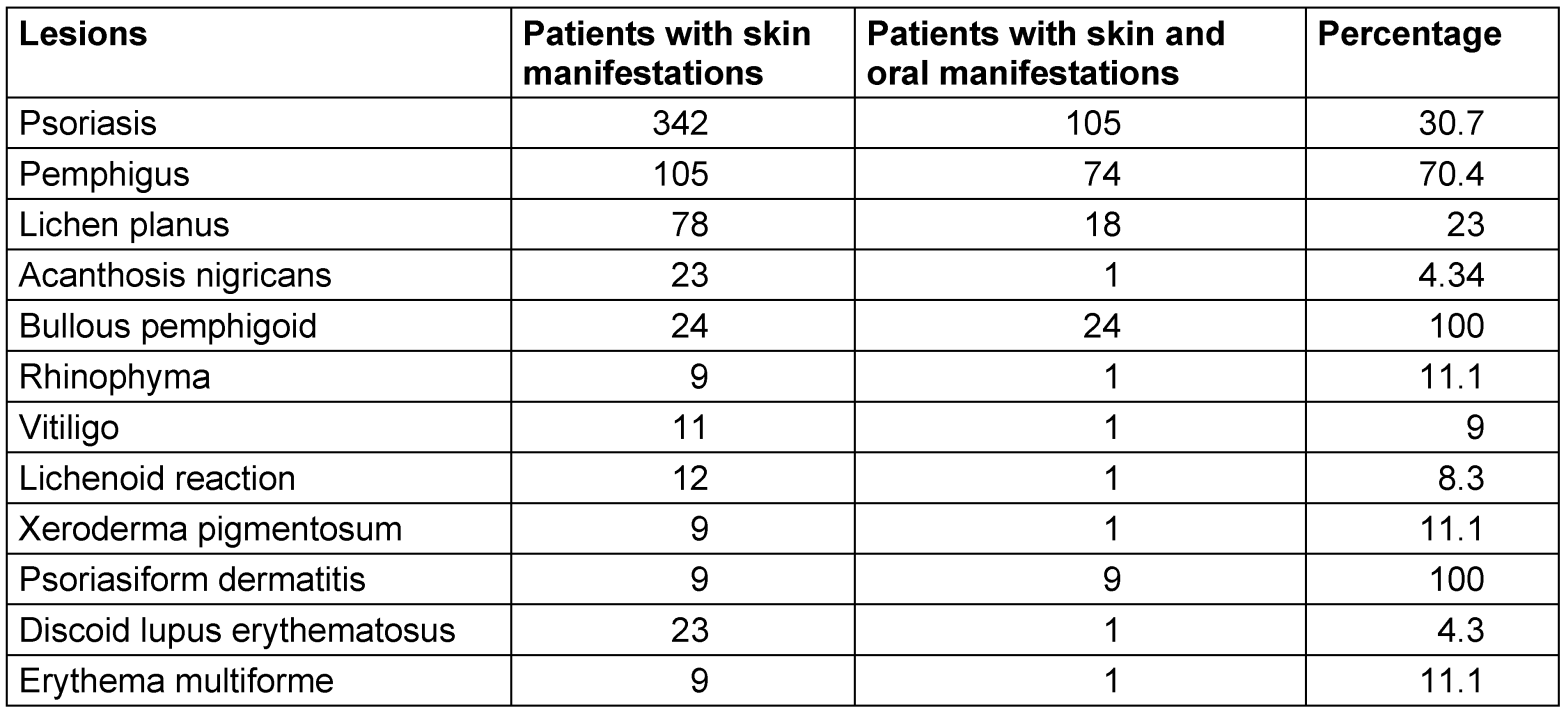
Percentage of oral mucosal lesions with various dermatological lesions

**Table 2:**
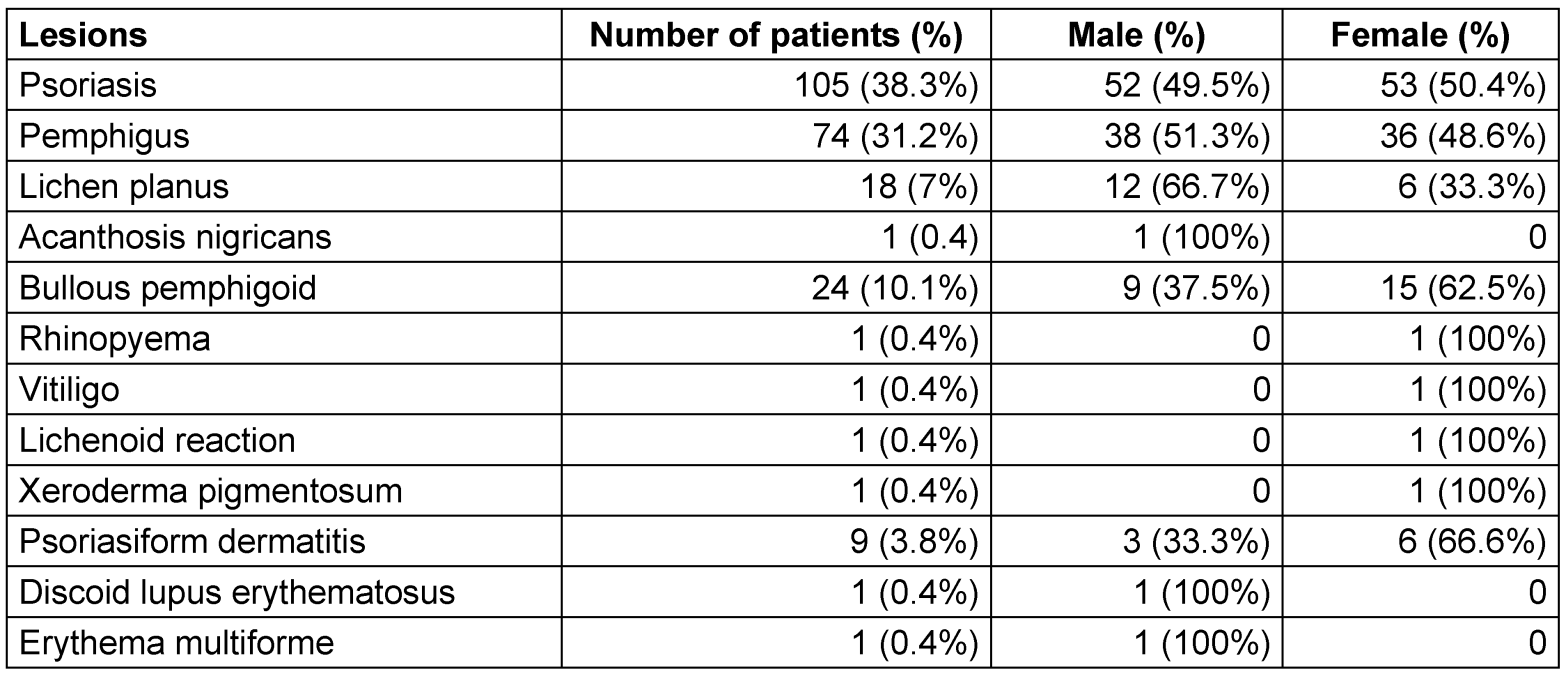
Gender distribution of dermatological diseases with oral mucosal lesions in the study population

**Table 3:**
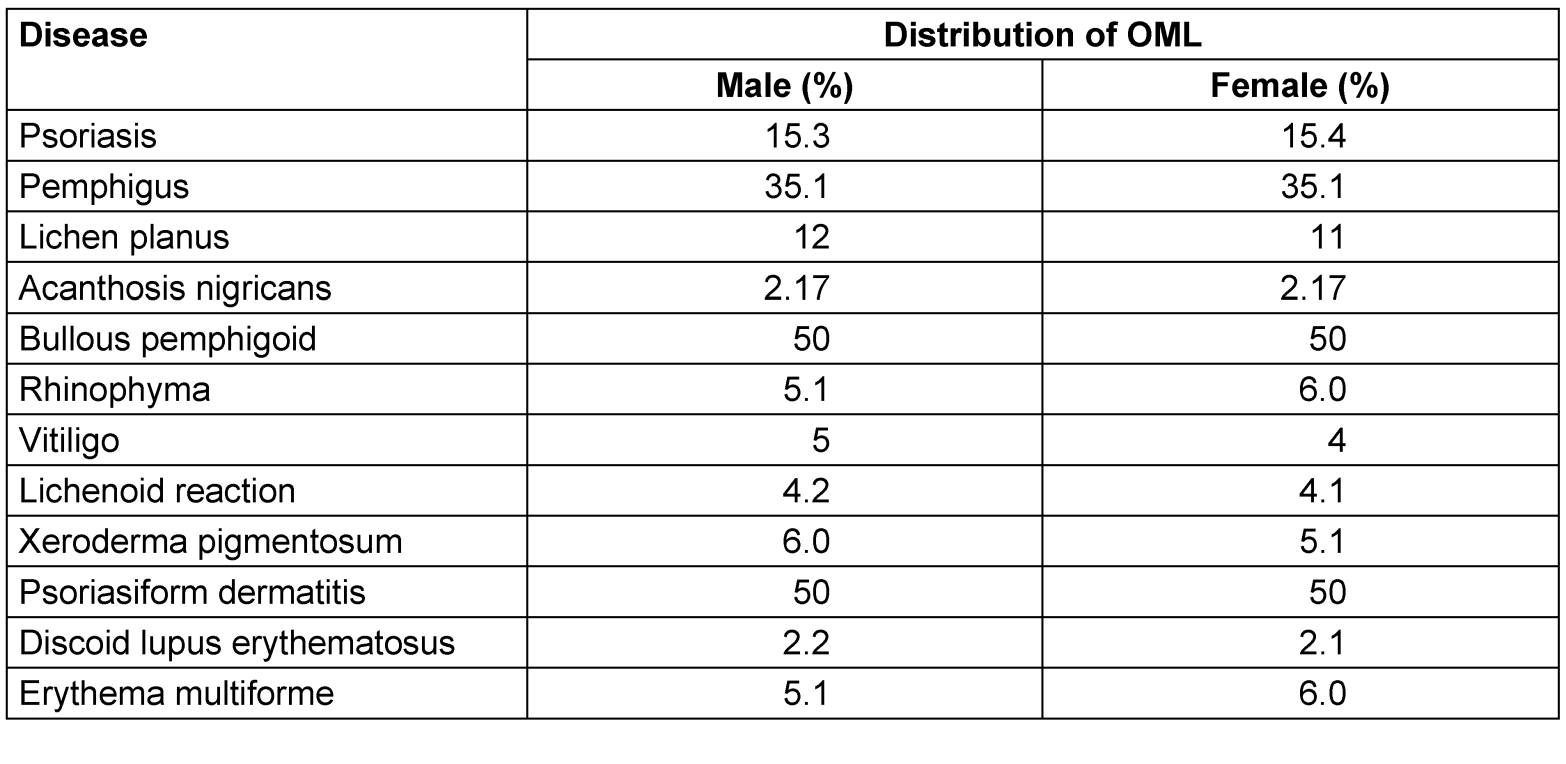
Gender distribution of oral mucosal lesions broken down into dermatological disease
